# Systemic lupus erythematous associated with multi-vessel spontaneous coronary artery dissection

**DOI:** 10.21542/gcsp.2023.24

**Published:** 2023-08-01

**Authors:** Dilesha D Kumanayaka, Ilsen Hernandez, Asrar Ahmad, Addi Suleiman

**Affiliations:** 1 Department of Internal Medicine, Saint Michael’s Medical Center/ New York Medical College, Newark, USA; 2 Department of Cardiology, Saint Michael’s Medical Center/New York Medical College, Newark, USA

## Abstract

Spontaneous coronary artery dissection (SCAD) is a rare cause of acute coronary syndrome (ACS), often associated with atherosclerosis. However, SCAD has been increasingly recognized as a distinct entity, especially in young females without traditional cardiovascular risk factors. We present a case of a 56-year-old female with systemic lupus erythematosus (SLE) who developed multivessel SCAD involving the right coronary artery (RCA) and ramus. The patient’s clinical presentation included typical chest pain, elevated troponins, and ST depressions on electrocardiography. Coronary angiography confirmed the presence of SCAD, classified as type 4 in the RCA and type 2 in the ramus. Prompt diagnosis and treatment resulted in a favorable prognosis. This case emphasizes the importance of considering SCAD in SLE patients presenting with ACS symptoms, particularly in younger women without evident cardiovascular risk factors. Early invasive coronary angiography is recommended for accurate diagnosis and timely management. SCAD can lead to significant complications and requires meticulous attention during angiographic procedures. Conservative management is often preferred, as most uncomplicated cases of SCAD heal spontaneously. Further research is needed to determine optimal treatment strategies and long-term outcomes for patients with SCAD, especially in the presence of underlying inflammatory conditions like SLE.

## Introduction

The majority of cases of acute coronary syndrome (ACS) in patients with systemic lupus erythematosus (SLE) are due to atherosclerosis^1^. Spontaneous coronary artery dissection(SCAD) is a rare cause of ACS in patients with SLE^1–7^. Spontaneous coronary artery dissection(SCAD) is defined as an intramural hematoma in the media of the vessel wall (false lumen) that flattens the true lumen, leading to blood flow obstruction and acute myocardial ischemia in the absence of trauma or iatrogenic causes^2^. The incidence of SCAD is suggested to be up to 1–4% among patients referred for angiography^4,5^. Multivessel SCAD is much rarer and occurs in 9–23% of described cases^6^. Females account for over 80% of SCAD cases^4,7^. The left anterior descending (LAD) artery is the most commonly affected coronary artery in SCAD^2,4–8^. During coronary angiography, SCAD findings can be graded into three types. Type 1 refers to the classic description of a longitudinal filling defect representing a radiolucent intimal flap. Contrast staining of the arterial wall with the appearance of a double lumen is often observed. Type 2 refers to diffuse long smooth tubular lesions (due to intramural hematoma) with no visible dissection plane, which can result in complete vessel occlusion. Type 3 is rare and mimics atherosclerosis; there is focal or tubular stenosis(<20 mm) but no atherosclerosis in the other coronary arteries^8^. Type 4 SCAD lesions are characterized by dissection, leading to abrupt total occlusion, usually of a distal coronary segment^9^. We report a case of MI secondary to multivessel SCAD involving the right coronary artery(RCA) and ramus in a young female with systemic lupus erythematosus (SLE).

## Case presentation

A 56-year-old female with a medical history of SLE (diagnosed by a rheumatologist in 2013 and treated with hydroxychloroquine) presented with intermittent, left-sided, pressure-like chest pain that radiated to her left shoulder for about 2-3 h. Each episode lasted for approximately 15 min, with no aggravating/relieving/precipitating factors.

She denied diaphoresis, palpitations, dyspnea, nausea/vomiting, headache, orthopnea, pedal edema, or paroxysmal nocturnal dyspnea. Patient was last pregnant more than 15 years ago. Patient was hypertensive (158/88), but other vital signs were normal. Lab work showed high sensitive troponins trended up from 3100 to 10700, with normal electrolytes, lipid panel and A1c.

**Figure 1.**
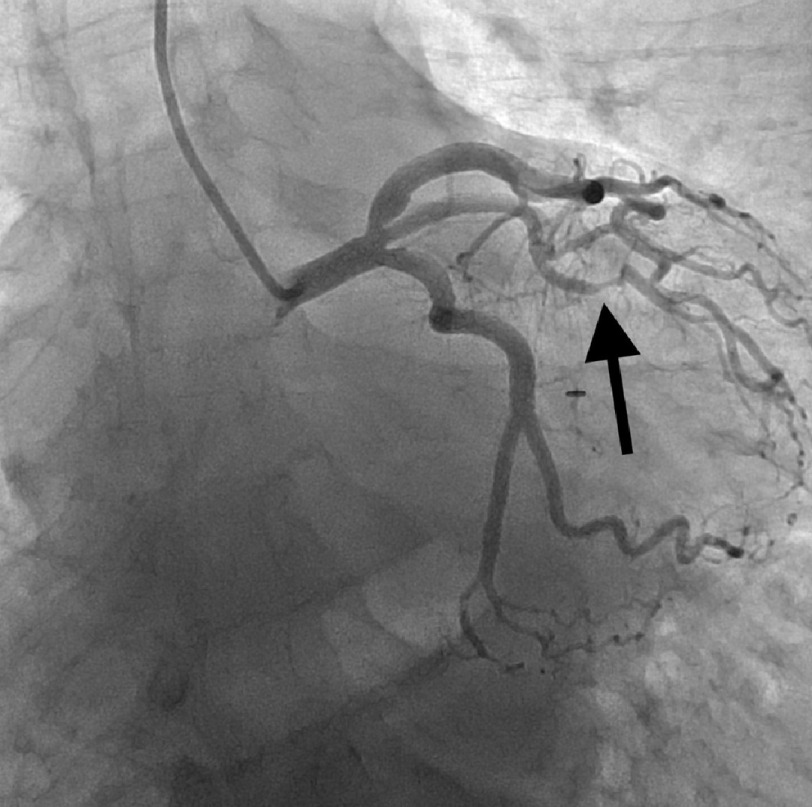
Coronary angiogram showing Ramus with SCAD type 2.

**Figure 2.**
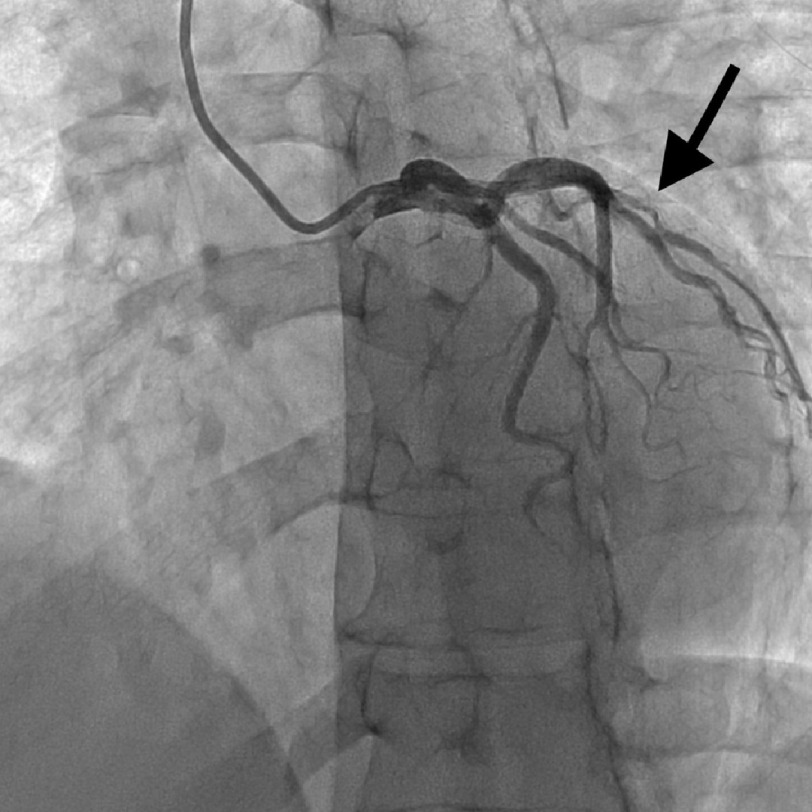
Coronary angiogram showing ramus with SCAD type 2.

**Figure 3.**
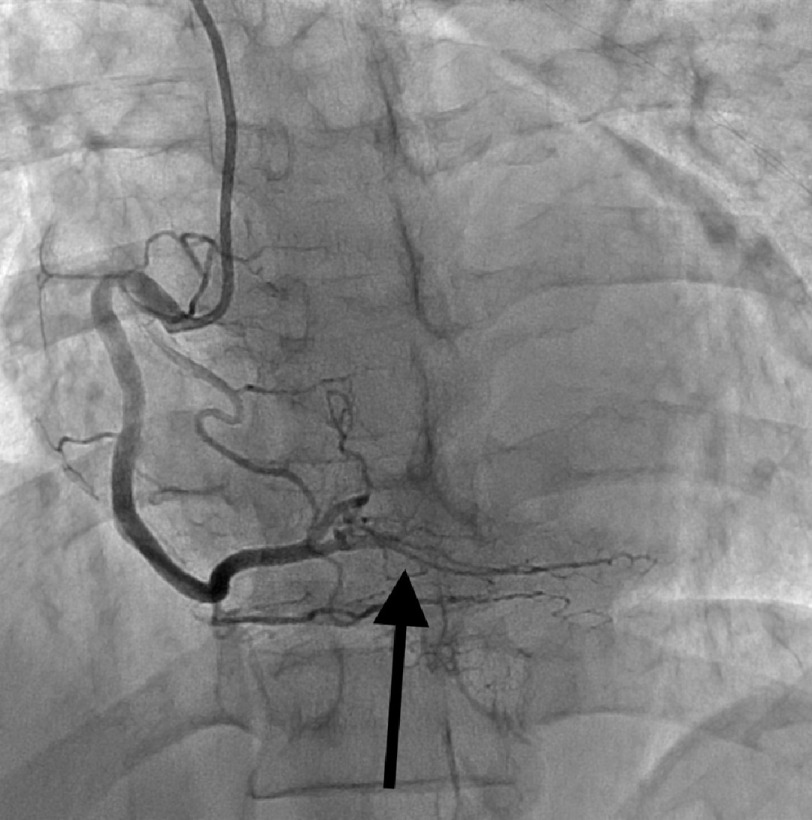
Coronary angiogram showing RCA with SCAD type 4.

EKG showed sinus rhythm, normal axis, and ST depressions in the lateral leads. Patient was loaded with ASA, statin and ticagrelor, and was then started on an unfractionated heparin drip as per ACS protocol. Transthoracic echocardiography showed global hypokinesia of the left ventricle with a mildly reduced ejection fraction of 40–45%. Left heart catheterization was immediately performed, which revealed SCAD in the RCA (type 4) and distal ramus (type 2), as shown in Figures 1–3 below. The patient was then monitored for a day and was discharged on dual antiplatelet therapy, beta-blocker, high-intensity statin, and ACE inhibitor with follow-up as an outpatient.

## Discussion

Multivessel SCAD is a rare but serious cause of ACS that requires prompt diagnosis and treatment. SCAD is a rare and under-reported phenomenon in SLE. The underlying mechanism of nonatherosclerotic SCAD remains uncertain^2,4–8^. One proposed mechanism of SCAD is the intimal tear hypothesis, which involves an intimal tear resulting in blood entering the intimal space from the endoluminal space, creating a false lumen filled with blood. The other mechanism is the medial hemorrhage hypothesis, which is thought to be due to rupture of the vasa vasorum, resulting in blood pooling within the intramural space, creating a false lumen filled with hematoma. Both result in the creation of a false lumen filled with an intramural hematoma^8^.

SCAD should be suspected in patients with SLE and ACS, particularly in younger women without evident cardiovascular risk factors. Early invasive coronary angiography is recommended in these patients^1,4,8^. Our patient did not have any predisposing factors other than SLE, but presented with typical chest pain, elevated troponins, and ST depressions on EKG. Timely management and diagnosis led to an excellent prognosis in our case. Our case highlights the importance of early, accurate diagnosis of SCAD which is essential to prevent complications and for specific treatment.

SCAD can result in significant complications such as myocardial ischemia and infarction, ventricular arrhythmias, and sudden cardiac death. Lack of angiographic recognition by clinicians is a major factor in under-diagnosis. SCAD likely results from factors that predispose arterial beds to injury, such as fibromuscular dysplasia, multiple pregnancies, systemic inflammatory diseases (SLE, Crohn’s disease, polyarteritis nodosa, and sarcoidosis), connective tissue disorders (Marfan’s syndrome, Ehler Danlos, and cystic medial necrosis), hormonal therapy, and coronary artery spasm^8^. During coronary angiography, SCAD findings can be graded into three types. Type 1 refers to the classic description of a longitudinal filling defect representing a radiolucent intimal flap. Contrast staining of the arterial wall with the appearance of a double lumen is often observed. Type 2 refers to diffuse long smooth tubular lesions (due to intramural hematoma) with no visible dissection plane, which can result in complete vessel occlusion. Type 3 is rare and mimics atherosclerosis; there is focal or tubular stenosis (<20 mm) but no atherosclerosis in the other coronary arteries^8^. Type 4 SCAD lesions are characterized by dissection, leading to abrupt total occlusion, usually of a distal coronary segment^9^.

Even though most clinicians use dual antiplatelet therapy for patients who are diagnosed with SCAD, it is unclear if the standard ACS pharmaceutical agents are beneficial for patients with SCAD not treated with stents. Considering the totality of evidence for aspirin in ACS and secondary prevention of CAD, aspirin appears reasonable for acute and long-term SCAD treatment if there are no contraindications, such as the risk of bleeding. P2Y12 receptor inhibitors such as clopidogrel are antithrombotic and are also reasonable in SCAD, in view of the intimal injury that can lead to thrombosis^8^.

## Conclusions

Our case was unusual in both patients’ prior diagnosis of SLE with no other risk factors and multivessel involvement of different types of SCAD, involving the less common coronary arteries (RCA and ramus). A differential diagnosis of SCAD should be included in all young female patients with ACS symptoms and/or signs, regardless of pregnancy status. Patients with SCAD, especially those with chronic inflammatory conditions, such as SLE, are thought to have more fragile coronary artery walls. Meticulous attention to angiographic techniques is required to avoid catheter-induced dissections. Conservative management is the preferred option, with spontaneous healing of the dissection in most uncomplicated cases of SCAD.
